# A cross-culture, cross-gender comparison of perspective taking mechanisms

**DOI:** 10.1098/rspb.2014.0388

**Published:** 2014-06-22

**Authors:** Klaus Kessler, Liyu Cao, Kieran J. O'Shea, Hongfang Wang

**Affiliations:** 1 Institute of Neuroscience and Psychology, University of Glasgow, Glasgow, UK; 2 Aston Brain Centre, Aston University, Birmingham, UK

**Keywords:** perspective taking, embodied transformation, line of sight, culture differences, gender differences, egocentric bias

## Abstract

Being able to judge another person's visuo-spatial perspective is an essential social skill, hence we investigated the generalizability of the involved mechanisms across cultures and genders. Developmental, cross-species, and our own previous research suggest that two different forms of perspective taking can be distinguished, which are subserved by two distinct mechanisms. The simpler form relies on inferring another's line-of-sight, whereas the more complex form depends on embodied transformation into the other's orientation in form of a simulated body rotation. Our current results suggest that, in principle, the same basic mechanisms are employed by males and females in both, East-Asian (EA; Chinese) and Western culture. However, we also confirmed the hypothesis that Westerners show an egocentric bias, whereas EAs reveal an other-oriented bias. Furthermore, Westerners were slower overall than EAs and showed stronger gender differences in speed and depth of embodied processing. Our findings substantiate differences and communalities in social cognition mechanisms across genders and two cultures and suggest that cultural evolution or transmission should take gender as a modulating variable into account.

## 1. Introduction

Some fundamental aspects of human social behaviour are shared with other species, whereas some aspects are uniquely human and typically involve representing and reflecting upon other's experiences and mental states, such as imagining another's perspective [1,2]. Perspective taking is a special and particularly interesting case in this context. Two different levels or types have been proposed based on developmental work by Flavell and co-workers [3] and cognitive work by Kessler & Rutherford [4] and Michelon & Zacks [5]. Importantly, one form seems to be uniquely human, whereas the other seems to be shared with other species.

**Figure 1.**
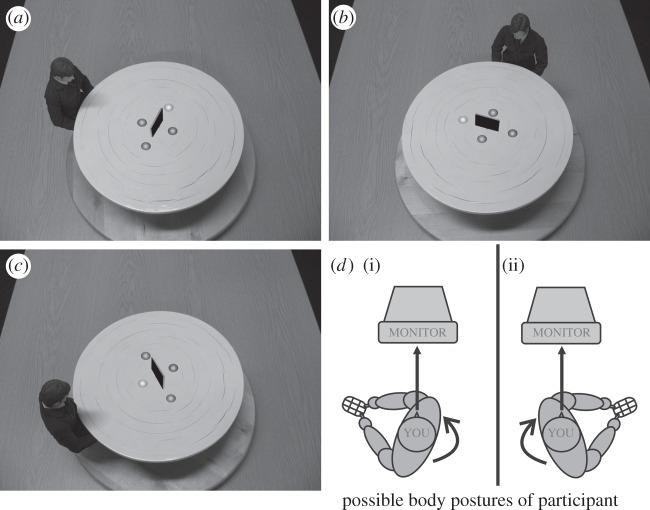
Stimuli and postures. Image (*a*) shows an example for a ‘left’ target from the avatar's perspective at 110° (clockwise angular disparity), image (*b*) shows an example for a ‘right’ target at 160° (anticlockwise), and image (*c*) shows an example for a ‘visible’ target at 60° (clockwise). In the figure, the target hemisphere is indicated by a brighter shade than the other three, whereas in the experiment colour stimuli were employed and the target changed colour from grey to red. Images (*d*(i)(ii)) show the two possible postures of the participant: body turned either (i) clock- or (ii) anticlockwise, while gazing straight ahead. The posture of the participant was therefore either congruent or incongruent to the direction of mental rotation on a particular trial.

Specifically, Flavell *et al.* [3] proposed that so-called level 1 perspective taking reflects understanding of *what* another can perceive, e.g. which objects are visible, which occluded to another person (see also figure 1), while level 2 involves mentally adopting someone else's point of view and understanding *how* the world is represented from this imagined perspective. A visuo-spatial example for level 2 perspective taking (VPT-2) would be telling a friend that she has an eyelash on her left cheek. This requires imagining ‘left’ and ‘right’ from our friend's perspective (cf. figure 1), thus involving a more complex mental operation than judging mere visibility (i.e. VPT-1). The two levels are mirrored in different developmental onsets [6–8], different response time (RT) patterns [5] and cross-species differences [2]. Apes, corvids and perhaps many other species seem capable of following gaze and of inferring what is visible or hidden from another's view in much the same way as humans [9–12]; in contrast, human aptitude for perspective taking extends far beyond that seen in other animals, although these higher forms of perspective taking may have phylo- and ontogenetic roots in their basic counterparts or in action control [13].

Indeed, VPT-2 has been linked to mentalizing and theory of mind on the one hand [14] and to embodied simulation of a body movement on the other [13] and is regarded as the more complex process of the two forms. This is evidenced by a later ontogenetic development [6–8], difficulties experienced by autistic children with VPT-2 but not with VPT-1 [14] and by phylogenetic differences, where primates and other species seem capable of certain forms of VPT-1 but not at all of VPT-2 [2].

However, primates [15,16] and other species [12,17–20] have been reported to *physically* align perspectives, e.g. align gaze direction with humans. Apes and ravens (*Corvus corax*) even deliberately change their position to be able to look around obstacles and share what a human experimenter can see [12,15,16]. This reflects the basic understanding that a physical or mental effort is sometimes necessary in order to understand someone else's view of the world [1]. We therefore hypothesized in our recent work that VPT-2 might have originated from deliberate physical alignment exhibited by apes and ravens [13]. We reasoned that if this was the case then VPT-2 would still be an ‘embodied’ process in form of a simulated body rotation, which was indeed supported experimentally [13] as explained below.

In terms of distinguishing VPT-1 and VPT-2 mechanistically, Michelon & Zacks [5] showed that where VPT requires visibility judgements only (VPT-1), it may be based on imagining the other's line-of-sight (LoS), which determines the relevant inter-object spatial relations between other, target and occluder; while VPT-2 in relation to left/right and other directional or visual judgements may involve mental self-rotation (SR) into the target perspective. Further, Kessler & Thomson [13] reported effects of congruence between participants’ body postures and the orientation of the target viewpoint (cf. figure 1). That is, VPT-2 was significantly faster and more accurate, when participants turned their body towards the target viewpoint (figure 1), confirming that SR for VPT-2 involves the simulation of a whole-body rotation into the target perspective (i.e. embodied SR: eSR). By contrast, body posture congruence had no effect on VPT-1 and simple visibility judgements [4], supporting the view that a simpler LoS mechanism is recruited in this case.

Substantial progress has been made in understanding the basic mechanisms of visuo-spatial perspective taking [4,5,13,21–24], yet, variability between individuals with respect to gender, culture, social skills, etc. has rarely been taken into consideration (for exceptions, see e.g. [14,25,26–29]). However, this would be essential for determining cultural and/or evolutionary contributions to this human capacity. For instance, strong cultural differences could indicate different cultural selection mechanisms, where different phenotypes might have different chances to proliferate, hence, further promoting a specific cultural environment in concordance with conformist transmission theory [30]. Thus, to increase our understanding of the variability across different groups of individuals, we compared VPT-1 and VPT-2 between males and females from two different cultural backgrounds: East-Asian (EA) versus Western (W).

### (a) Differences between genders and cultures

Kessler & Wang [26] recently reported that social skills (as measured with the ‘social skills’ subscale of the Autism-spectrum Quotient [31]) predicted the strength of embodiment (body posture effect, cf. Kessler & Thomson [13]) during VPT-2 in a W sample. Gender proved to be another critical factor and females were more embodied, yet slower at high angular disparities than males (revealing larger slopes). Thus, W systemizers (males/low social skills) do not seem to ‘embody’ another's perspective as deeply as empathizers (females/high social skills), but seem to be faster. It appears that empathic depth is traded for higher speed, which could be either a sign of strategic flexibility or, alternatively, a lack of social skill.

In cultural environments where a social orientation towards others rather than the self is actively encouraged (e.g. EA-culture; cf. [32,33]), individuals might generally become more adept at imagining other's viewpoints and perspectives resulting in more efficient (faster, more accurate) use of strongly embodied strategies or, alternatively, in more flexibility regarding the deployment of minimal resources. That is, highly skilled perspective takers might possess the flexibility to rotate a reduced body schema, e.g. head/eye based in contrast to whole-body based [34], making their eSR process less effortful. Hence, the question is whether a strongly other-oriented cultural background might somewhat paradoxically result in a pattern similar to W systemizers (EA-group: flexible, fast, minimally embodied) or whether it would resemble more strongly the pattern of W ‘embodiers’ (EA-group: empathic, deeply embodied)—yet faster, owing to practice. Further, if gender could be mapped onto a systemizer–empathizer dimension across cultures [35], one would expect particularly effective VPT-2 mechanisms in EA-females.

Initial evidence that the postulated cultural differences are indeed reflected in different patterns of VPT and, importantly, in different strengths of egocentric bias, was reported by Wu & Keysar [28,36]. In a ‘visual world’ communication game [37], participants moved objects within a grid according to a ‘director's’ verbal instructions. Some objects were occluded from the director's view and only visible to the participant. In contrast to an EA-group, W participants were more strongly affected by competitor objects which the director could not see, revealing egocentric bias. However, a recent reanalysis of the time course of these eye-tracking data suggests that the other-centred bias in the EA-group was the result of a late correction process of an initial egocentric interference pattern similar to W culture [36]. Hence, ego- versus other-centred cultural biases in perspective processing still remain to be understood in detail.

In this study, we set out to elucidate how an egocentric bias in Ws and an other-centred bias in EAs, respectively, might influence the basic mechanisms of VPT. Kessler & Rutherford [4] observed that in a W sample, ‘visible’ responses were accomplished significantly faster than ‘occluded’ responses (‘visibility advantage’), which is plausible given that visible targets are directly within the LoS of the avatar and do not require consideration of the occluder (also [38]). Importantly, Kessler and Rutherford found the strongest advantage for visible over occluded responses at 60°, i.e. at the maximum overlap between the avatar's and the egocentric LoS (figure 1), reflecting an egocentric influence on processing of the other's perspective. Visible targets were also closest to the participant at this angular disparity while occluded targets were furthest away: at 60° Ws might actually encode visibility in relation to themselves rather than to the other's LoS. An egocentric bias could also explain why the visibility advantage fades away at 160°: the closeness of the ‘occluded’ target to the participant in contrast to the distance of the ‘visible’ target (figure 1) might cancel out an advantage for visible targets from the other's perspective. By contrast, if EA participants would exhibit a different pattern, i.e. no such bias towards maximum overlap between avatar and egocentric LoS, then the notion would be supported that EA-culture discourages an egocentric bias in VPT-1 processing.

### (b) This study

We hypothesized that, in principle, an embodied mental SR (eSR) process would be employed for VPT-2, whereas a line-of-sight (LoS) mechanism would subserve VPT-1 across cultures and genders. However, an other-oriented, collectivistic and holistic processing style was expected to favour EAs in terms of overall speed for VPT-1 and VPT-2. More specifically, we also expected visible versus occluded effects to distinguish between a more egocentric (W group) versus a more other-oriented (EA group) bias in VPT-1. For VPT-2, we expected EAs to be more efficient (faster), yet the depth of embodiment (magnitude of posture effect) could either reflect a stronger urge to empathize (enhanced posture effect) or more flexibility (reduction) in the amount of body schema required for embodied mental simulation. Finally, our cultural comparison included gender as a potentially moderating factor [26]. If gender could be mapped onto a systemizer–empathizer dimensional space across cultures [35], we expected particularly effective VPT-2 mechanisms in EA females.

## 2. Material and methods

### (a) Participants

All participants were enrolled at university or had previously received a university education. None of our participants was simultaneous or infant bilingual of English (or any other W language) and Mandarin (or any other Chinese dialect) according to standard definitions [39]. Participants received payment of £5/¥30 for completing the experiment. The W sample consisted of 64 European participants (33 females; mean age = 22.36, s.d. = 3.2) all of whom were tested at the University of Glasgow and were predominantly reading Psychology (22) and other Social Sciences, including Education, Languages, Philosophy and Economics (46 in total), while a minority (18) were reading Law or Natural Sciences (Chemistry, Biology, Zoology, Neuroscience and Medicine), Statistics, Mathematics, Computer Science or Engineering. The Chinese sample also consisted of 64 participants (33 females; mean age = 22.36, s.d. = 1.7). Thirty-four participants were tested at Wuhan University, China, and the majority were also reading Psychology (18) or other Social Sciences, including Economics, Arts and Philosophy (26 in total), while a minority (8) were reading Natural Sciences or Engineering. The remaining 30 Chinese participants were tested at the University of Glasgow, within the first three months of their arrival in the UK, and 23 were reading Social Sciences, including Psychology, Education and Economics, while seven were reading Natural Sciences, Engineering or Accountancy. A *χ*^2^ test revealed that the distribution of reading Social versus Natural Sciences (including Law, Accounting, Engineering) did not differ significantly (*χ*² = 0.37; *p* = 0.54) between the Chinese (49 : 15) and the W (46 : 18) sample. All procedures complied with the ethical codes of conduct of the American Psychological Association, British Psychological Association and the declaration of Helsinki.

### (b) Stimuli and apparatus

The employed VPT tasks and stimuli were adopted from Kessler & Rutherford (Experiment 1, [4]). In all stimuli, an avatar was presented seated at a round table shown from one of six possible angular disparities (60°, 110°, 160° clockwise and anticlockwise; cf. figure 1). The stimuli were coloured photographs (resolution of 1024 × 768 pixels), taken from an angle of 65° above the plane of the avatar and table. The stimulus table contained four grey spheres (placed around an occluder, cf. figure 1). In each trial, one of the spheres turned red indicating this sphere as the target. From the avatar's viewpoint, the target could be visible/occluded (VPT-1) or left/right (VPT-2) and participants were asked to make a judgement according to the avatar's perspective. In English, the instructions were: ‘try to place yourself in the other person's perspective and press the corresponding key for whether the target is left or right or whether it is visible or occluded’. For the Chinese sample, we generated a translation that was expected to be processed in the same way as the English version. Liyu Cao (co-author) was the experimenter for the Chinese samples at Wuhan and Glasgow University and ensured that the instructions were understood in an identical fashion to the English version.

Stimuli were presented and responses were recorded using E-Prime v. 2.0. Participants sat on a swivel chair and responded to the stimuli using a wireless computer mouse, which was secured to a padded plastic board on their lap. Viewing distance of the participant from the computer screen and the resulting visual angle was varied between groups of participants: one W and one EA sample (*n* = 34, 17 females in each) were stimulated at a visual angle of 22.16° × 13.85° (at 1024 × 768 pixels screen resolution), while another W and another EA sample (*n* = 30, 16 females in each) were stimulated at a visual angle of 32.7° × 18.4°. We varied visual angle as an alternative route for potentially tapping into culture-related differences in cognitive processing [40]. However, anticipating our results, the manipulation of visual angle did not significantly impact on our data.

### (c) Procedure and design

Every participant received 16 mini-blocks, eight for each VPT task presented in an alternating sequence. The first two mini-blocks consisted of six practice trials each and enabled participants to familiarize themselves with the experimental stimuli. The remaining 14 experimental mini-blocks contained 24 trials each. Task instructions were given at the beginning of each block by indicating whether it was a left/right or a visible/occluded block and reminding participants of the correct key mappings. For the VPT-1 task, participants were required to press the left mouse button with their left forefinger to indicate that the red sphere was ‘visible’ or the right mouse button with their right forefinger to indicate that the red sphere was ‘occluded’. For the VPT-2 task, participants pressed the left button for a ‘left’ and the right button for a ‘right’ target.

Note that Kessler & Rutherford [4] reported one experiment (Experiment 1) that used key-press responses and a second experiment (Experiment 2) that used vocal responses. We found the same pattern of results across the two experiments disregarding response modality. It is important that the basic RT pattern was replicated with vocal responses as these do not depend on spatially mapped key-presses and therefore do not induce spatially incongruent stimulus-response mappings [41]. Thus, if our current study would replicate the pattern reported in Kessler & Rutherford [4], then we could be confident that the findings were not primarily due to spatial incompatibilities in stimulus-response mappings.

Most importantly, conforming to our previous studies [4,13], participants’ body posture was randomly varied across trials. At the beginning of each trial, participants were instructed to sit in either a clockwise or counter-clockwise posture (according to an instruction picture shown on screen, cf. figure 1), while keeping their head facing towards the screen. In other words, their body posture could be either congruent or incongruent with a clockwise or anticlockwise direction of mental SR.

After adopting the indicated posture for a given trial, participants pressed both mouse buttons to initiate the trial. A fixation cross was displayed for 500 ms before the stimulus picture appeared, and participants were required to respond as quickly and as accurately as possible.

The resulting 2 × 2 × 2 × 2 × 2 × 3 mixed design included three between-subjects factors with two levels each: culture (EA versus W), gender (male versus female) and visual angle (small versus large), as well as three within-subjects factors: task (VPT-1 versus VPT-2), body posture (congruent versus incongruent posture) and angular disparity (60° versus 110° versus 160°, collapsed across clockwise and anticlockwise disparities). The complete dataset is available at the Economic and Social Research Council Data Store: http://store.data-archive.ac.uk:80/store/collectionEdit.jsp?collectionPID=archive:957.

## 3. Results and discussion

Our analysis focused on RTs (for correct responses only) because both VPT tasks were performed close to ceiling level in terms of accuracy by all groups (i.e. less than two mistakes on average across all conditions). Individual RT medians for each condition were used for the purpose of reducing distortions caused by outliers [4,13]. The sphericity assumption was violated in relation to model terms involving angular disparity (Mauchly's tests *p* < 0.05), hence, MANOVA analysis was employed that does not assume sphericity (see [13, p. 77] for discussion). We followed up on significant interactions in the full design MANOVA by means of separate MANOVAs for VPT-1 and VPT-2, respectively (indicated in brackets), as well as by means of planned comparisons of simple contrasts.

**Figure 2.**
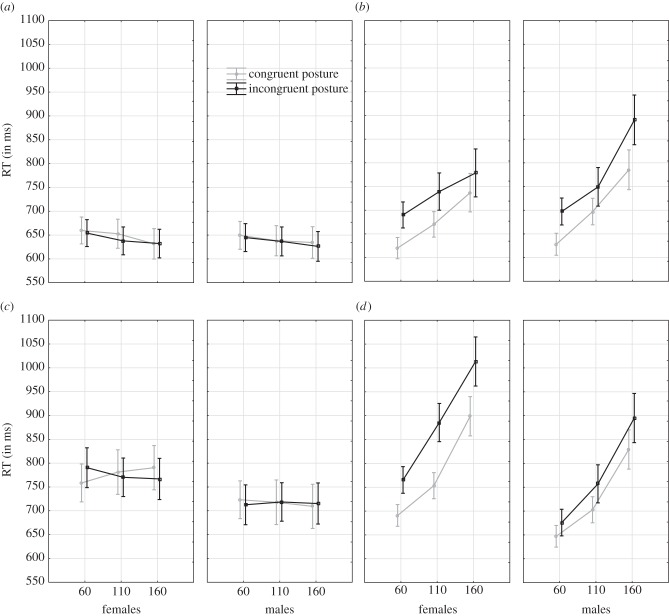
Interaction between task × angular disparity × posture × gender × culture. Panel (*a*) shows the findings for VPT-1 in the EA group, panel (*b*) for VPT-2 in the EA group, panel (*c*) for VPT-1 in the W group and panel (*d*) for VPT-2 in the W group.

RTs were subjected to the described 2 × 2 × 2 × 2 × 2 × 3 mixed design (visual angle, gender, culture, task, posture, angular disparity) MANOVA. ‘Visual angle’ did not reach significance and did not interact significantly with any of the other factors (all *p* > 0.1). The main effects of culture (*F*_1,120_ = 9.2, *p* = 0.003, 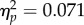), task (*F*_1,120_ = 25.3, *p* < 0.00001, 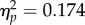), posture (*F*_1,120_ = 79, *p* < 0.00001, 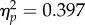) and angular disparity (*F*_2,119_ = 52.1, *p* < 0.00001, 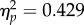) reached significance. Significant interactions between task and posture (*F*_1,120_ = 108.7, *p* < 0.00001, 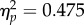) as well as between task and angular disparity (*F*_2,119_ = 74.4, *p* < 0.00001, 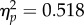) revealed stronger posture and angular disparity effects for VPT-2 compared with VPT-1 (figure 2). Culture- and gender-specific modulations were also evidenced by significant interactions between: angular disparity × culture (*F*_2,119_ = 4.3, *p* = 0.015, 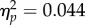), posture × gender × culture (*F*_1,120_ = 5.6, *p* = 0.02, 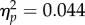); task × posture × gender × culture (*F*_1,120_ = 5.9, *p* = 0.016, 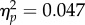); task × angular disparity × posture × gender (*F*_2,119_ = 4.34, *p* = 0.0152, 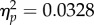) and task × angular disparity × posture × gender × culture (*F*_2,119_ = 4.33, *p* = 0.0153, 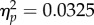). The latter five-way interaction modulated the other lower level interactions and is best understood by considering figure 2.

### (a) The global pattern: similarities across groups

The pattern of RT results shown in figure 2 confirms previous observations that VPT-1 and VPT-2 are subserved by qualitatively distinct mechanisms [4,5,21]. However, these two mechanisms seem to be comparable, in principle, across cultures and genders as we observed the same basic pattern reported by Kessler & Rutherford [4] for both cultures and genders: VPT-1 RTs (figure 2*a*,*c*) did not increase with angular disparity and were not affected by posture in the same way as VPT-2 (figure 2*b*,*d*). For the latter, RTs increased for all groups across angular disparities (60°, 110°, 160°) and a congruent posture was processed faster than an incongruent posture (figure 2*b*,*d*; all *p* < 0.00012). The similarity in basic patterns across groups (culture, gender) was reflected by the strongest effect sizes 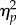) for the interactions between task × posture and task × angular disparity reported above. Furthermore, the interactions between task × angular disparity and task × posture reached significance for each group (EA females, EA males, W females, W males), when tested separately (all *p* < 0.0001). Nevertheless, this basic common pattern for each VPT task was modulated differently by culture and gender, although, it is important to point out that effect sizes for model terms involving the between-subject factors culture and gender were much smaller than those for the within-subject factors angular disparity and posture (and interactions with task). Hence, in statistical terms the commonalities seem to outweigh the differences.

### (b) Modulations by culture and gender

The EA group was faster than the W group across both VPT tasks (i.e. main effect of culture), but for VPT-1 this was reflected by generally faster RTs across all angular disparities (main effect of culture in a VPT-1 only MANOVA: *F*_1,120_ = 11.9, *p* < 0.001, 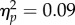), while for VPT-2 RTs differed only at high angular disparities (interaction between angular disparity × culture in a VPT-2 only MANOVA: *F*_2,119_ = 3.15, *p* < 0.05, 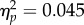). At 60°, we did not observe any significant differences for VPT-2 between the groups (all *p* > 0.05), indicating that groups were comparable in their baseline speed for judging left and right (at 60°, the target configuration was most closely aligned with the egocentric view, hence, we suggest it can be regarded as a baseline indicator).

The RT pattern for the two VPT tasks was further modulated by gender and culture (i.e. significant five-way interaction reported above), where W females were slowest overall for both tasks (also compared to W males), yet, where W females were also the strongest ‘embodiers’ overall. W females revealed significant posture congruence effect for VPT-2 across all angular disparities (*p* < 0.00001) yet also for VPT-1 at 60° (*p* = 0.01), however, with numerically reversed effects for VPT-1 at 110° and 160° (both *p* > 0.05). For a VPT-1 only analysis (cf. figure 2*a*,*c*), this resulted in a significant interaction between angular disparity × posture × gender × culture (VPT-1 MANOVA: *F*_2,119_ = 3.4, *p* < 0.05, 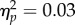), because none of the other three groups (W males, EA females, EA males) revealed any significant posture effects for VPT-1 (all *p* > 0.1).

By contrast, for VPT-2 all groups revealed significant posture effects (all *p* < 0.00012), yet, these effects were also modulated by culture and gender (interaction between posture × culture × gender in a VPT-2 only MANOVA: *F*_1,120_ = 6.9, *p* < 0.01, 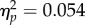). As can be observed in figure 2, the W group revealed significant gender differences with respect to speed and posture congruence (both *p* < 0.004), replicating Kessler & Wang ([26]; see Introduction). By contrast, no significant gender differences were observed for the EA group in terms of main effects of speed and posture congruence (both *p* > 0.1).

### (c) Gender-specific differences between cultures for VPT-2

The strongest cross-cultural differences for VPT-2 were observed between females (figure 2*b*,*d-*left graph): W females were slower than EA females (Wilk's *λ* (3,118) < 0.00001, *p* < 0.00001), but W females were more embodied, showing a stronger posture congruence effect (*F*_1,120_ = 5.8, *p* = 0.017). A more detailed analysis revealed (figure 2*b*,*d*, left graph) that the posture effects at 160° and 110° differed significantly (both *p* < 0.05) but did not reach significance at 60° (*p* = 0.82). This seems to indicate that females from both cultural backgrounds start off at 60° with a comparable amount of embodiment, yet, EA females seem to be more flexible in adjusting their level of embodiment, allowing for faster processing of the other's perspective at high angular disparities.

The special role of EA females as the most effective perspective takers is corroborated by a significant interaction between posture × gender for the EA group at 160° (*F*_1,62_ = 6.21, *p* = 0.015). EA males were more embodied (but slower), revealing a contrasting effect to the W group. Overall, males seemed to be more comparable across the two cultures in terms of speed and posture congruence effects (figure 2*b*,*d*). EA males only revealed a significantly stronger posture congruence effect than W males at 60° (*F*_1,120_ = 4.25, *p* = 0.041) (figure 2*b*,*d*, right graph).

We predicted that the other-oriented socio-cognitive style in EA culture (cf. [32,33]) would augment the effectiveness of VPT-2, which would result in faster overall processing times, while the strength of embodied processing was an open issue. EAs could have been more similar to W systemizers (flexible, fast, but minimally embodied) or to W ‘embodiers’ (empathic, deeply embodied)—yet fast, owing to practice. Replicating the Kessler & Wang [26] finding for the W group (females being slower but more embodied) provided us with the opportunity to tackle this issue. The data seem to support the first possibility, as both EA groups were faster but less embodied than the W females and did not differ significantly overall from the W males.

Nonetheless, it is important to highlight that both EA genders showed comparable posture effects to W females but stronger posture effects than W males at 60°. This could suggest that both genders in the EA group—but EA females in particular—were more flexible with respect to the (reduced) amount of body schema they employed for mental SR at high angular disparities (110°, 160°). Our findings could suggest that at high angular disparities (especially 160°), EA females might be more practiced and therefore more flexible than the other three groups in rotating a reduced body schema, resulting in the fastest RTs overall for EA females, thus, underlining EA females as the most effective ‘mental self-rotators’ in our sample.

### (d) Ego- and other-centred biases in ‘visible’ versus ‘occluded’ judgements (VPT-1)

In concordance with our hypotheses, we included ‘response type’ (‘visible’ versus ‘occluded’) as a factor in a MANOVA for VPT-1 trials only (design factors: visual angle, gender, culture, posture, angular disparity, response type). To re-iterate, for a W group Kessler & Rutherford [4] had reported the strongest advantage for visible over occluded responses at 60° indicating an egocentric bias in Ws (i.e. at 60°, the overlap between the avatar's and the egocentric line-of-sight is maximal; also, visible targets are closest to the participant, while occluded targets are furthest away). If the current EA group would exhibit a different pattern, it could suggest an other-oriented bias in EA culture for VPT-1 processing.

In addition to the significant main effect of culture (*F*_1,120_ = 11.9, *p* < 0.001, 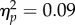) and interaction between angular disparity × posture × gender × culture (*F*_2,119_ = 3.4, *p* < 0.05, 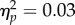) already discussed in the previous sections, the current analysis also revealed a main effect of response type (*F*_1,120_ = 7.5, *p* < 0.01, 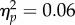), with ‘visible’ being faster than ‘occluded’, and an interaction between response type × angular disparity × culture (*F*_2,119_ = 4.1, *p* < 0.05, 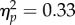).

**Figure 3.**
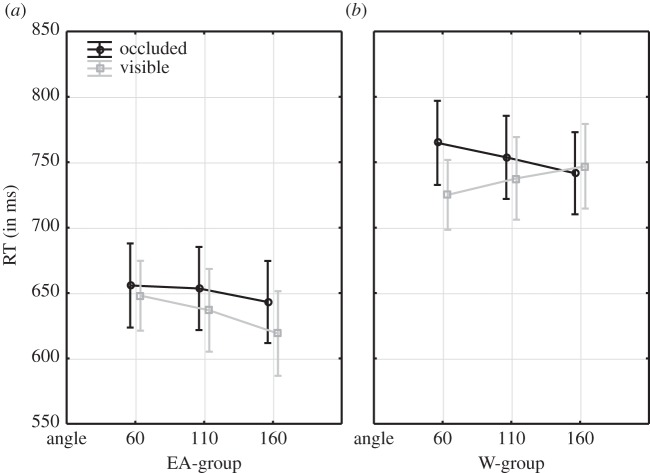
Interaction between angular disparity × response type × culture for VPT-1 trials only. Hence, response type refers to ‘visible’ versus ‘occluded’ judgements.

First, the results for the W group (figure 3*b*) successfully replicated the pattern reported by Kessler & Rutherford [4]: ‘Visible’ were particularly faster than ‘occluded’ responses (visibility advantage) at the lowest angular disparity of 60° (*F*_1,120_ = 11.3, *p* < 0.001; all other *p* > 0.05), corroborating the notion of an egocentric bias in the W group, because visible targets were also closest to the participant (at 60°), while occluded targets were furthest away. At 60°, Ws might actually encode visibility in relation to themselves rather than in relation to the other person's LoS, ‘assimilating’ the other's perspective into the egocentric view. This could also explain why the visibility advantage fades away at 160°: the closeness of the ‘occluded’ target to the (W) participant in contrast to the distance of the ‘visible’ target might conflict with the visibility advantage from the other's perspective, cancelling each other out. In both cases (60° and 160°), an egocentric bias in Ws can explain the presence versus absence, respectively, of a visibility advantage.

Second, in contrast to the W group, the EA group revealed the strongest response type effect, or visibility advantage (visible < occluded) at the largest angular disparity of 160° (*F*_1,120_ = 7, *p* < 0.01; all other *p* > 0.05), where the avatar's LoS was maximally misaligned with the egocentric perspective, suggesting a different processing style in the EA group (figure 3). It seems that EA participants were processing the other's perspective most effectively when it was most different from the egocentric viewpoint at 160°, in other words, when any ‘confusion’ from the egocentric view could be most effectively suppressed. At 60°, however, where the perspectives largely overlap, the egocentric perspective seems to rather interfere with effective processing of the other's perspective (instead of the other's perspective being ‘assimilated’ into the egocentric view as proposed for Ws). This resulted in significantly increased RTs at 60° compared with 160° in the EA group (*F*_2,119_ = 4.8, *p* = 0.01)—an effect not observed for Ws (*F*_2,119_ = 0.03, *p* = 0.97)—and in a lack of difference between ‘visible’ versus ‘occluded’ judgements at 160° (possibly owing to a ceiling effect).

Thus, our results support the notion of an other-centred bias in EA culture. This interpretation is also in line with the significant main effect of culture (EA faster than the W group) and with the observation that RTs significantly decreased across angular disparities in the EA group but not in the W group. Our results emphasize fast and holistic processing of another's perception of the world (VPT-1)—while effectively suppressing the egocentric view—in the EA group and in contrast to the W group. Finally, we did not find significant effects of gender in either culture, suggesting that for simpler VPT-1 perspective taking the observed cultural biases might be gender independent.

## 4. General discussion

The obtained pattern of results replicated previous findings and supported our current hypotheses. In line with our previous research, we confirmed that, in principle, both cultures and genders employ the same eSR process for VPT-2, and the same LoS mechanism for VPT-1, as indicated by the strongest effect sizes for model terms involving angular disparity, posture and their interactions with task. This suggests that the use of these basic mechanisms could generalize beyond the currently investigated samples. However, it is important to point out that our samples are limited in their generality with participants being recruited from the student population in both cultures and with a further bias towards Psychology/Social Sciences. In the light of these limitations, further studies are necessary to corroborate our conjecture that the human species as a whole may have developed the capacity for mental simulation of complex relationships in the world, and in this particular case, for simulating another's view of the world as exemplified by VPT-2. This is in line with a proposition by Tomasello *et al*. [2] that humans not only differ in aspects of computational ability from other primates but crucially in their motivation to share their experience and view of the world with others in socially meaningful ways.

It is noteworthy that our samples from both cultures followed a clear-cut separation between eSR for VPT-2 and LoS for VPT-1, respectively. Although larger and more diverse samples (e.g. larger age, profession and cultural range), will be necessary for an ultimate conclusion, the present outcome is quite striking, because one could have expected that an other-centred cultural environment might encourage the adoption of effortful but more empathic embodied processing, i.e. eSR, for all types of perspective judgements, including VPT-1. Instead, the EA group also used the minimum-effort mechanism, LoS, whenever the task permitted (i.e. ‘visibility’ judgements) and even displayed higher proficiency than the W group in terms of speed, potentially indicating that this strategy might generalize to a wide range of situations outside the laboratory. Crucially, LoS processing was modulated differently in the two cultures, revealing an other-centred bias in the EA group and an egocentric bias in the W group. That is, in replication of Kessler & Rutherford's [4] finding in a W sample, the W group's ‘visibility advantage’ (‘visible’ < ‘occluded’ RTs) was biased towards maximal overlap between avatar and their own perspective (i.e. at 60°), while the EA group oriented most effectively towards the other's perspective when it was maximally distinct from the self (i.e. at 160°).

Furthermore, for the W group, we confirmed [26] that females were slower for VPT-2 but more strongly embodied than males. By contrast, genders in the EA group were more comparable overall, differing significantly only at 160° angular disparity in the VPT-2 task, where males were slower yet more embodied (i.e. opposite pattern to W culture). EA females in particular were highly efficient perspective takers in the VPT-2 task, showing significant embodiment but fast processing at the same time: at the highest angular disparity of 160°, they were significantly fastest overall. As a consequence, the strongest cultural differences regarding VPT-2 performance were found between females, where EA females were faster but less embodied than W females (at 110° and 160°). EA participants in general were faster overall than W participants across both VPT tasks, while revealing significant posture effects for VPT-2 that were significantly stronger than for W males at 60°, and which could reflect a bias towards embodied, but highly efficient and flexible other-oriented processing in EA culture compared with W culture. All-in-all, conforming to our expectations, EA participants were the more effective perspective takers and this was particularly pronounced for EA females.

The cultural differences across both VPT tasks are in agreement with notions of cultural and conformist transmission [30], where specific values—or biases in the current context—are maintained via social learning of culture-specific behaviours and which seems to be supported by natural selection of specific genotypes that further promote conformist behaviour [30]. Although speculative at the current stage, EA and W culture may have followed slightly different conformist transmission trajectories, with the former promoting more strongly other-oriented values and favouring selection of individuals with high social skills such as VPT-2 efficiency, while the latter promoting more egocentric values and individuals with strong individualistic and/or leadership qualities. Our findings add to the growing body of evidence that this may have been the case [28,32,33,36]. Finally, based on our findings, we further speculate that cultural transmission may have been different for the two genders across both male-dominant cultures, with females being specifically required to become excellent perspective takers—in both cultures, but even more so in EA culture—while males were only required to achieve a minimum level of VPT-2 proficiency, thus, possibly remaining more comparable across cultures. Although the details of our reasoning are highly speculative at the current stage, especially in the light of our limited samples, our findings nevertheless emphasize that investigations of culture-specific evolution of social values and behaviours should take gender as a potentially modulating factor into account.

## Notes

We obtained ethical approval from the University of Glasgow Ethics Committee and each participant gave their informed consent in writing.

## Data accessibility

The complete dataset is available at the ESRC Data Store: http://store.data-archive.ac.uk:80/store/collectionEdit.jsp?collectionPID=archive:957.
